# A Facile Thermal-Treatment Route to Synthesize ZnO Nanosheets and Effect of Calcination Temperature

**DOI:** 10.1371/journal.pone.0103134

**Published:** 2014-08-05

**Authors:** Naif Mohammed Al-Hada, Elias B. Saion, Abdul Halim Shaari, Mazliana A. Kamarudin, Moayad Husein Flaifel, Sahrim Hj Ahmad, Salahudeen A. Gene

**Affiliations:** 1 Department of Physics, Faculty of Science, University Putra Malaysia, Serdang, Selangor, Malaysia; 2 School of Applied Physics, Faculty of Science and Technology, Universiti Kebangsaan Malaysia, Bangi, Selangor, Malaysia; Washington State University, United States of America

## Abstract

A facile thermal-treatment route was successfully used to synthesize ZnO nanosheets. Morphological, structural, and optical properties of obtained nanoparticles at different calcination temperatures were studied using various techniques. The FTIR, XRD, EDX, SEM and TEM images confirmed the formation of ZnO nanosheets through calcination in the temperature between 500 to 650°C. The SEM images showed a morphological structure of ZnO nanosheets, which inclined to crumble at higher calcination temperatures. The XRD and FTIR spectra revealed that the samples were amorphous at 30°C but transformed into a crystalline structure during calcination process. The average particle size and degree of crystallinity increased with increasing calcination temperature. The estimated average particle sizes from TEM images were about 23 and 38 nm for the lowest and highest calcination temperature i.e. 500 and 650°C, respectively. The optical properties were determined by UV–Vis reflection spectrophotometer and showed a decrease in the band gap with increasing calcination temperature.

## Introduction

Nanoscale particles possess several unique properties such as large surface areas, unusual adsorptive properties, surface defects and fast diffusivities. For that, this kind of nanomaterials is quite appealing in industrial and commercial applications. Recently, metal oxide nanoparticles are attractive subject of continuous scientific research and have been deeply investigated because of their physical and chemical properties as well as for their wide range of applications including sensors [1], semiconductors [2], magnetic materials [3], catalysts [4], optoelectronic materials [5], medical [6] and environmental remediation [7].

Zinc oxide (ZnO), which is a direct wideband gap semiconductor compound of the II–VI family, has a wurtzite crystalline structure at ambient conditions with hexagonal unit cell and two lattice parameters, *a* and *c*. The structure is composed of two alternating interpenetrating hexagonal closed packed (HCP) planes stacked layer-by-layer, in which each consists of oxygen anion tetrahedrally coordinated with four zinc cations at the corners of the tetrahedron along the threefold *c*-axis. The tetrahedral coordination of ZnO gives rise to the noncentrosymmetric structure which exhibits sp^3^ covalent-bonding.

Zinc Oxide (ZnO) semiconductor compound has a direct band gap of 3.37 eV at room temperature and a large excitation binding energy (60 meV), which make them promising photonic material for several applications, especially in the solid state light sources and detectors in the blue and UV spectral ranges [8]. When zinc oxide approaches nanoscale sizes accompanied by intriguing properties due to quantum confinement effect [8] as compared to bulk counterparts, its usage can further expand to cover applications as in optical devices including blue-violet and UV-light emitting diodes [9], laser diodes [10], biosensors [11], piezoelectric transducers, solar cells [12], transparent electrodes, electroluminescent devices and photocatalytic materials [13]. Moreover, because it has been chemically and optically stable with offering low toxicity, its use as a fluorescent label for bio-imaging in medical application has been anticipated [14].

Various methods have been previously reported on the synthesis of ZnO nanosheets with improved chemical and physical properties, such as solvothermal method [15] sol–gel technique [16], precipitation method [17], hydrothermal growth (HTG) method [18], microwave assisted method [19], hydrothermal method [20], anodization method [21], thermal decomposition and reduction method [22]. Further methods also have been reported to synthesis ZnO nanparticles such as Microemulssion method [23]–[26], microwave method [27], thermal evaporation [28], mechanochemical synthesis method [29], spray pyrolysis [25] and chemical vapour deposition [30]. However, most of the methods are difficult to employ in a large scale production due to the complicated procedures involved, longer reaction times, high reaction temperatures, and the involvement of toxic reagents and by-products in these synthesis methods.

In this study, thermal-treatment method was used to synthesize zinc oxide nanosheets from an aqueous solution containing only zinc nitrate, polyvinyl pyrrolidone (PVP) and deionized water. This method has the advantages of being simple, less expensive, has no unwanted by-products, and is environmentally friendly [31], [32]. The effect of calcination temperature on the structural and optical properties of ZnO nanosheets was also investigated and discussed in detail.

## Experimental Section

### 1. Materials

Poly (vinyl pyrrolidone) (PVP M_W_ = 29000 g/mol) was purchased from Sigma Aldrich and utilized as a capping agent which is anticipated to stabilize the nanoparticles and hence reduce their agglomeration. Zinc nitrate reagent, Zn (NO_3_)_2_ •6H_2_O (M_W_ = 297.47 g/mol) and deionized water were supplied by Acros Organics SIGMA and used as metal precursor and solvent, respectively. The chemical materials were above 99% in purity and used without further purification.

### 2. Synthesis of nanosheets

The PVP solution was made by dissolving 3 g of PVP powder in 100 ml of deionized water at a temperature of 70°C in a water-bath and allowed to stir for 2 hrs. Later, zinc nitrate of 0.2 mmol was added into 100 ml PVP solution and the mixture was stirred continuously for 2 hrs until a colourless solution was obtained. No precipitation of materials was observed in the solution before it was poured in Petri dish and dried at 80°C for 24 hrs. The resulting solid was crushed for 30 min in a mortar to form powder. The powder was subsequently divided into five portions and each portion was placed in a crucible alumina to be calcined at a specific temperature for 3 hrs in order to crystalize the nanosheets and decompose the organic compounds.

### 3. Characterization

Several techniques were employed to investigate the synthesised ZnO nanosheets. The structure of ZnO nanosheets has been examined by X-ray diffractometry (XRD Shimadzu model 6000) using Cu Kα (0.154 nm) as a radiation source to generate diffraction patterns from the crystalline powder samples at ambient temperature in 2θ range of 10^°^–90^°^. Infrared spectra (280–4000 cm^−1^) have been recorded using Fourier transform infrared spectrometer (FTIR Perkin Elmer model 1650) to confirm the removal of capping agent after calcination. Basically, both XRD and FTIR results were used to prove the formation of crystalline ZnO powder at different calcination temperatures. The morphology, particle size, particle size distribution and the homogeneity of the nanosheets were determined using transmission electron microscopy (JEOL TEM model 2010F UHR) with an accelerating voltage of 200 kV. Moreover, UV–vis spectrophotometer (Shimadzu model UV-3600) was used in order to evaluate the optical properties of the samples at room temperature in the range of 200–800 nm.

## Results and Discussion

### 1. Formation mechanism of ZnO nanosheets

**Figure 1:**
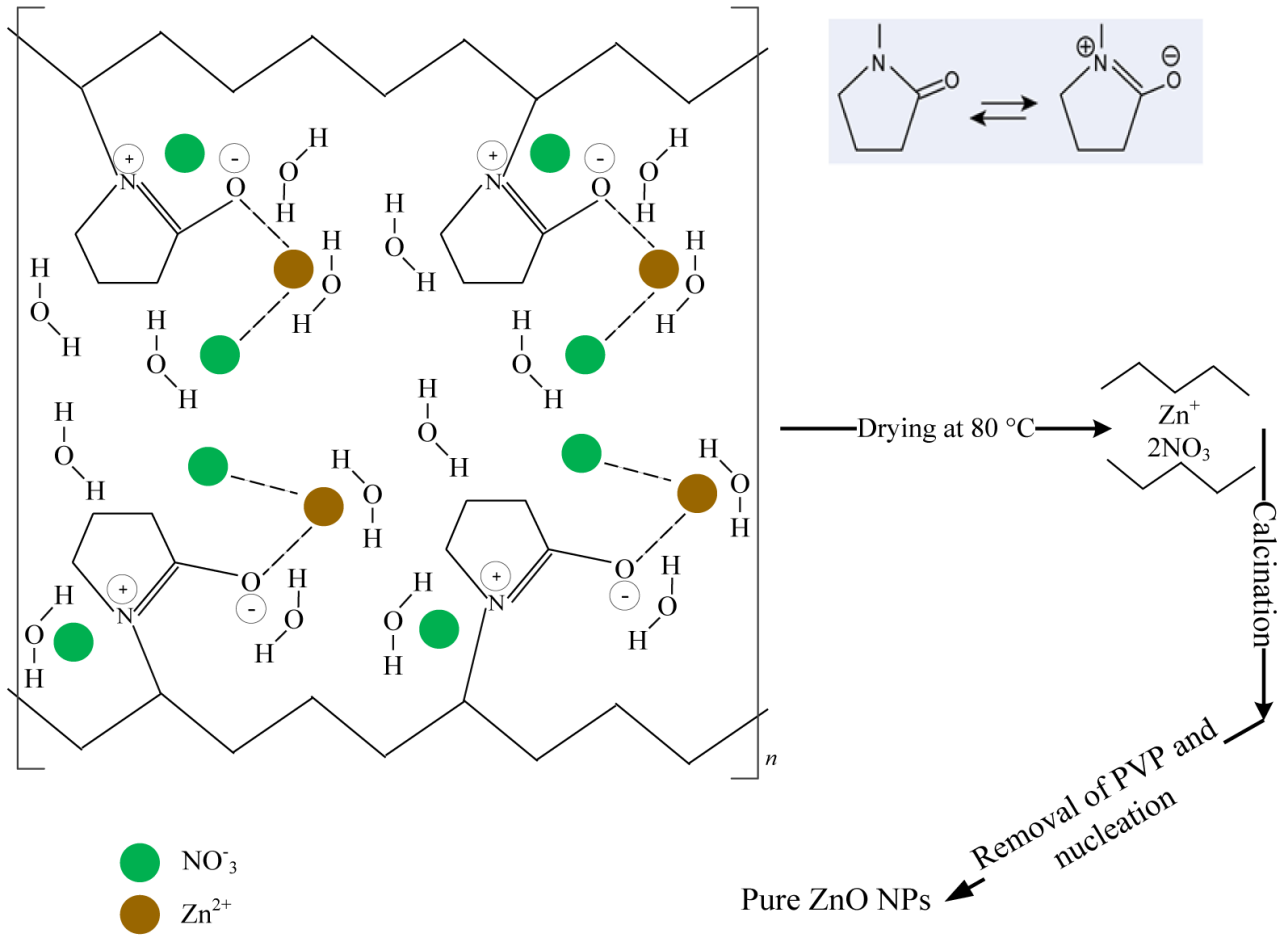
A proposed mechanism of the interaction between metallic ions and PVP.

**Figure 1** shows a proposed interaction mechanism between Zn metallic ions and PVP. The zinc nitrate Zn (NO_3_)_2_ •6H_2_O was added to the aqueous solution of PVP and deionized water (DI) in which it was completely dissolved. In fact, while mixing the solution, the metallic ions subdued an entrapment with amine group in the polymeric chains through ionic-dipole interactions. This was followed by a mobility congealment of the metallic cations in the polymer cavity as a result of water removal when the mixture was dried. After calcination being applied, the PVP content and undesired anions were completely removed from the sample and gradually high purity ZnO nanosheets begun to conform with increasing calcination temperature. It is believed that PVP agent has played a role in the nucleation of ZnO nanosheets and has been a controller for the particle's size growth [33].

### 2. TGA-DTG measurements

**Figure 2:**
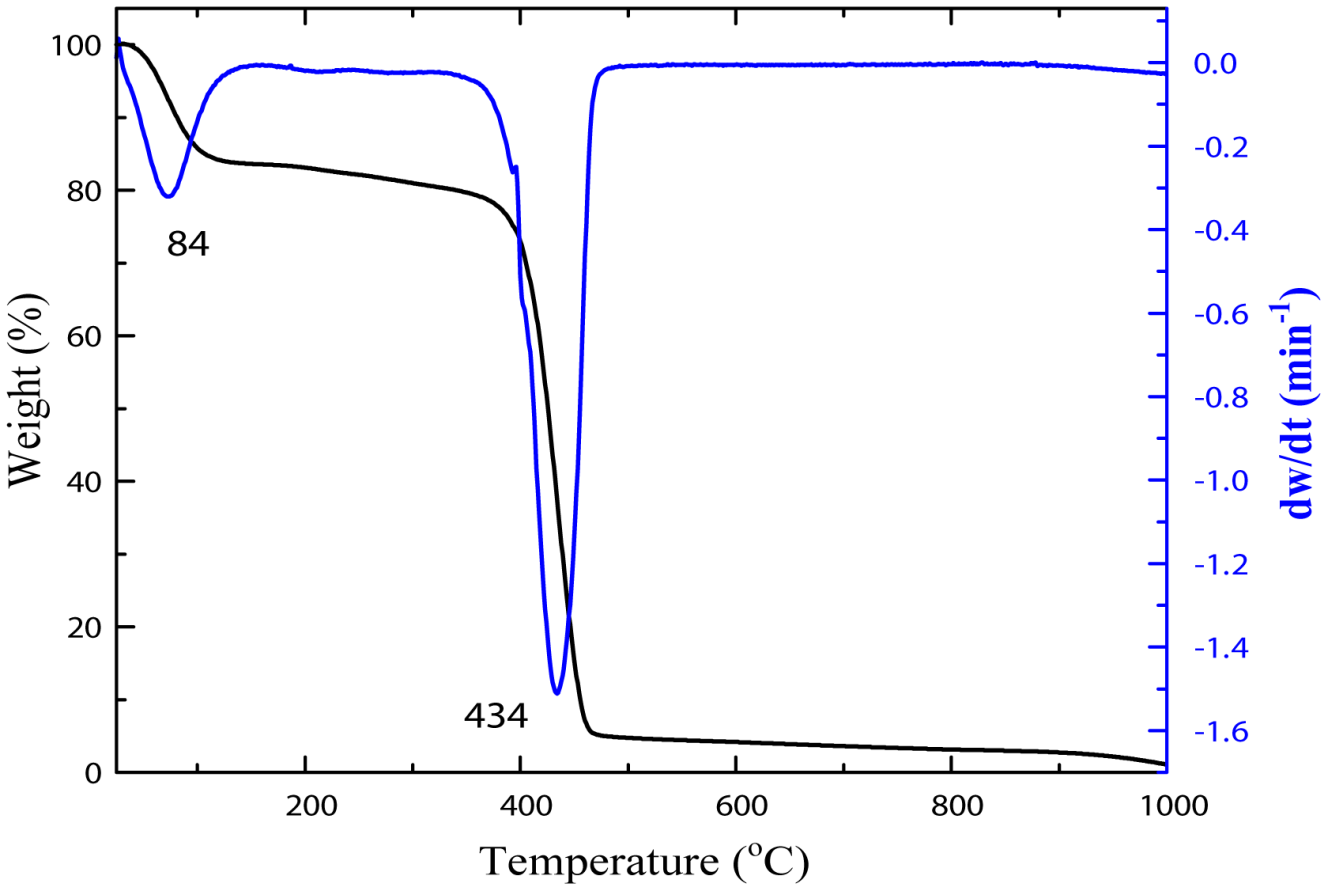
Thermogravimetric (TG) and thermogravimetric derivative (DTG) curves for PVP at a heating rate of 10°C/min.

Thermogravimetric analysis and its derivative form (TGA-DTG curves) were used so as to determine the appropriate starting temperature for the calcination process. **Figure 2** shows the thermogram of percentage weight change as a function of temperature for the sample before calcination. It is evident that the sample exhibited two step degradation processes. The first step shows insignificant weight loss occurred at an initial degradation temperature of 84°C, which is attributed to the trapped moisture in the sample. The second one demonstrates a maximum weight loss occurred at a temperature of 434°C, which corresponds to almost complete decomposition of PVP in the sample. At 485°C, the weight loss change as a function of temperature became insignificant as most of the PVP content in the sample turned to carbonaceous products and hence leaving pure metal oxide nanosheets of the sample as the final residue [33].

### 3. Phase and elemental composition analysis

**Figure 3:**
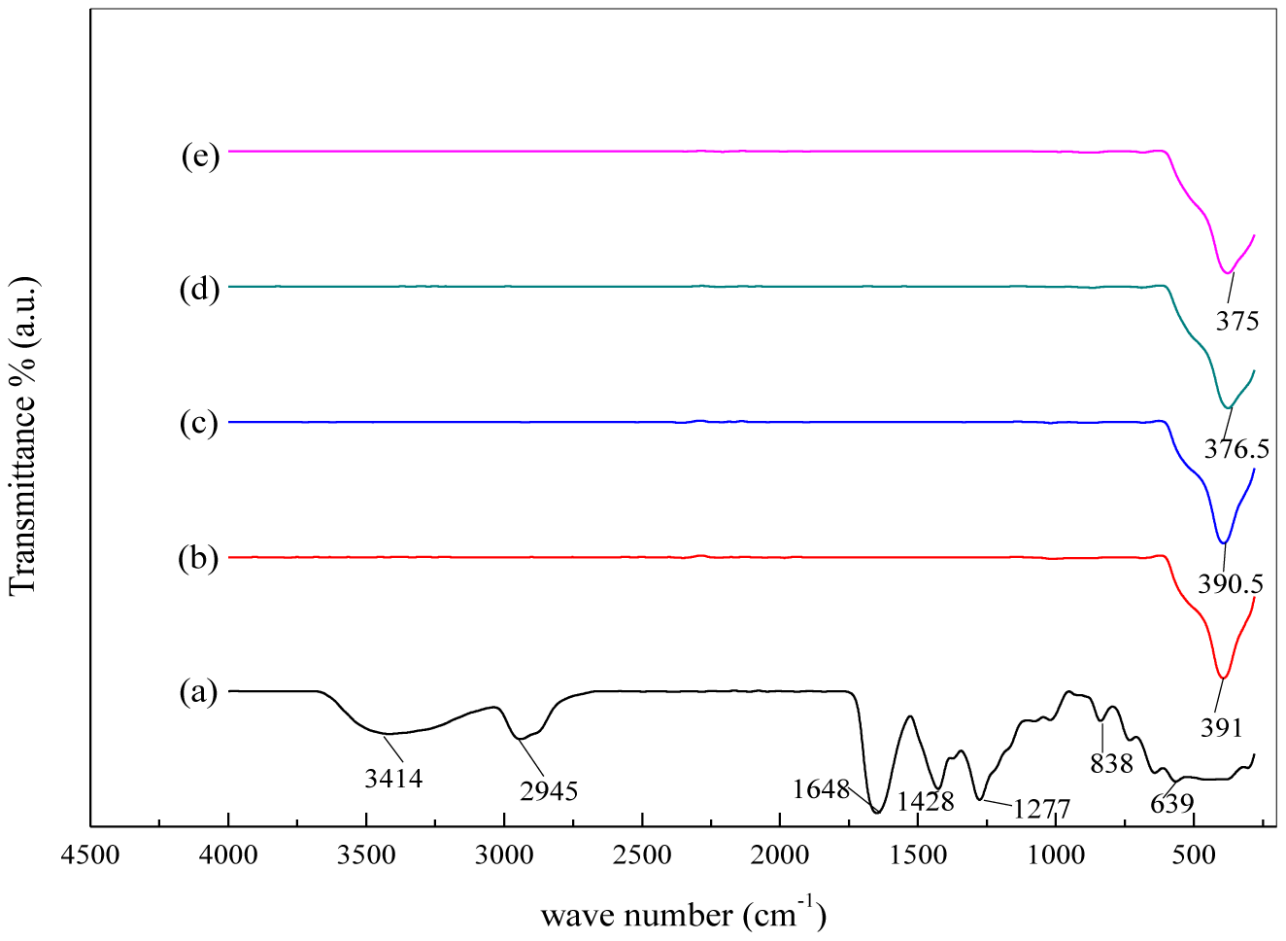
FTIR spectra of (a) PVP and ZnO nanosheets at (a) 30, (b) 500, (c) 550, (d) 600, and (e) 650°C in the range of 280–4500 cm^−1^.

FTIR spectroscopy helps analysing multi-component systems and provides necessary information pertaining the material's phase composition and types of interactions existing amongst various kinds of polymers. In our study, FTIR measurement was employed to determine the appropriate calcination temperature at which pure crystal nanosheets conform with no organic agent trace being detected. This further can be evidenced through investigating the interaction between ZnO nanosheets and PVP at different calcination temperatures using TEM images. The FTIR spectra show the organic and inorganic contents of the sample before and after being calcined over the wave number range of 280–4000 cm^−1^. **Figure 3** **(a)** exhibits all absorption peaks that are attributed to the PVP and ZnO nanosheets. Before calcination the absorption peaks at wave number of 3414, 2945 and 1646 cm^−1^ were assigned to N–H, C–H and C = O stretching vibrations, respectively. Further, the absorption peak found at 1428 cm^−1^ was ascribed to C–H bending vibration originated from methylene group, while 1277 cm^−1^ was associated with C–N stretching vibration. Finally, 839 and 639 cm_−1_ were corresponding to the vibrations occurred due to C–C ring and C–N = O bending [34]. The calcination at 500°C resulted in the disappearance of broadband absorption peaks that belong to the organic composition of PVP as evident in **Figure 3** **(b,c,d,e)**. The vibrational spectra of ZnO nanosheets samples were observed as absorption at the wave number of 391, 385, 376 cm^−1^ and 375 cm^−1^ calcined at 500,550,600°C and 650°C, respectively. The presence of single absorption peak and the observed shift in the wave number for ZnO nanosheets spectra with increasing calcination temperature imply that high purity ZnO nanosheets have been obtained using thermal-treatment method.

**Figure 4:**
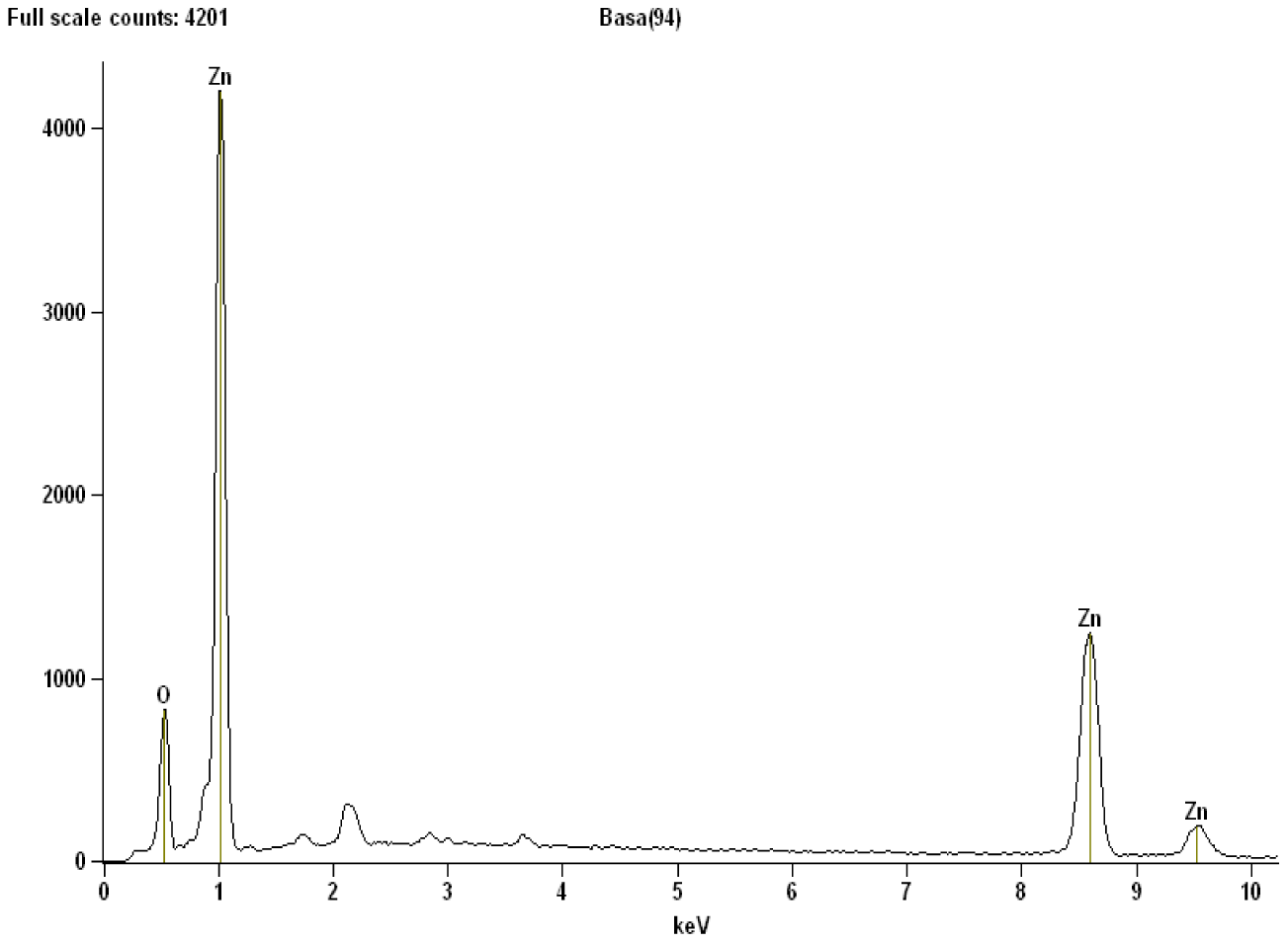
The EDX spectrum of the ZnO nanosheets clacined at 500°C.

The elemental composition of the nanosheets sample formed by thermal-treatment technique was determined using energy dispersive X-ray (EDX) spectroscopy. EDX spectrum of ZnO nanosheets calcined at 500°C is illustrated in **figure 4**. From the spectrum, the Zn and O elements are vividly present in the prepared sample as shown by their respective peaks. The recorded atomic percentages of Zn and O were approximately 50.13% and 49.87%, respectively. This result indicated that the final product is pure ZnO nanosheets.

### 4. Structural analysis

**Figure 5:**
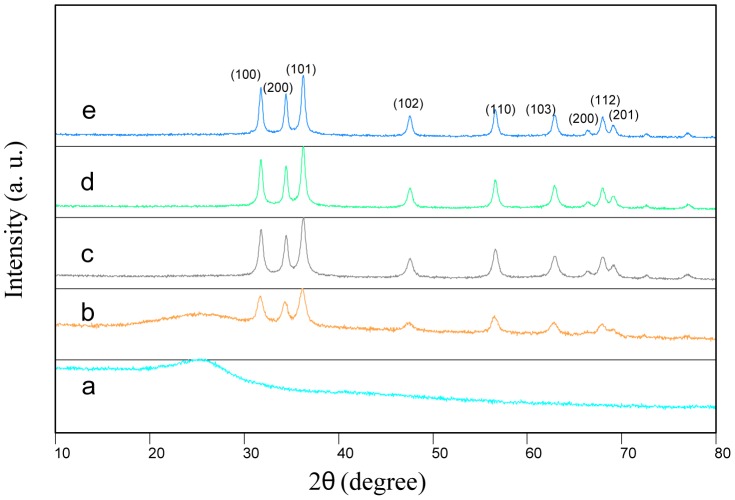
XRD patterns of zinc oxide nanosheets with different calcination temperatures of (a) room temperature, (b) 500, (c) 550, (d) 600, and (e) 650°C.

**Figure 5** shows typical XRD patterns of the samples before and after calcination. A broad spectrum exhibited by the sample before calcination indicates that the dried samples demonstrated an amorphous behaviour although physically appeared to behave like crystalline materials. For the calcined samples at 500°C and above, the spectrum shows sharper and narrower diffraction peaks, implying that the crystalline ZnO nanosheets formation has been established. Further, higher values of calcination temperatures were observed to enhance the crystallinity of the ZnO nanosheets by increasing the intensity of the peaks and removing the undesired small peaks appeared at lower calcination temperatures. This crystallinity enhancement with increasing calcination temperatures is stemmed from the increment of the crystalline volume to surface ratio, as evidenced by TEM images, which occurred due to particle size enlargement. From **Table 1**, it is also apparent that the d-spacing has decreased with increasing calcination temperatures. The position of the Bragg's lines of ZnO nanosheets was used to determine the interplaner spacing (d), which in turn used to index the diffraction peaks. The existence of multiple diffraction peaks of (100), (200), (101), (102), (110), (103),(200),(112) and (201) ) in the diffraction patterns suggests that the ZnO samples have a typical hexagonal structure referring to JCPDS card no. 36–1451 data [32], [35]. The samples' crystallite sizes were found to range from 24 to 41 nm, which were calculated for the most intense peak (101) using the Scherrer's equation as given below;
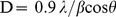
(1)where *D* is the crystallite size (nm), β is the full width of the diffraction line at half of the maximum intensity i.e. (101) which is measured in radians, λ is the X-ray wavelength of Cu Kα = 0.154 nm and θ is the Bragg's angle [1].

**Table 1:** Briefing of the structural and optical properties of synthesized ZnO nanosheets at different calcination temperatures.

Temperature (°C)	2θ±0.001	FWHM±0.001	d (nm)	D_XRD_ (nm)	D_TEM_ (nm)	Eg (eV)
500	36.22	0.36	2.4799	24	23±2	3.26
550	36.23	0.32	2.4796	27	26±3	3.25
600	36.37	0.28	2.4756	31	30±2	3.24
650	36.39	0.21	2.4744	41	38±4	3.23

### 5. Morphological examination

**Figure 6:**
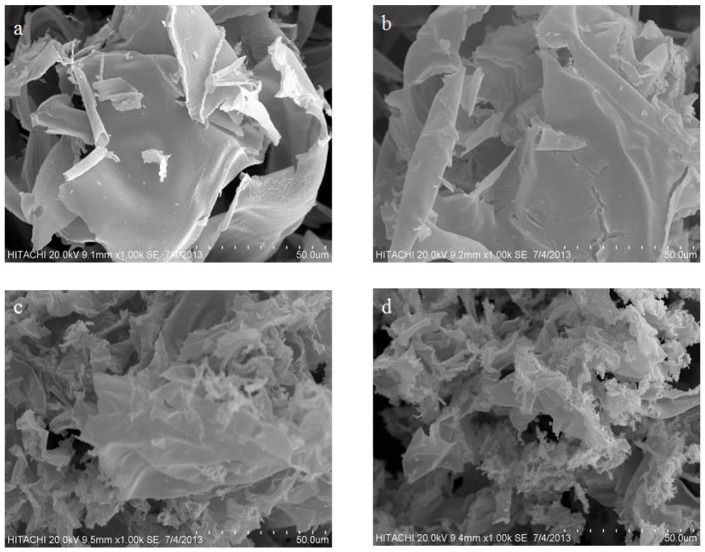
SEM images of ZnO nanosheets at calcination temperatures of (a) 500 and (b) 550, (c) 600 and (d) 650°C.

The surface morphology of ZnO nanosheets were demonstrated using scanning electron microscopy (SEM). **Figure 6**, shows SEM images of ZnO nanosheets and the calcination temperature's effect. The results are in agreement with previously reported literature [19], [22], [36]. **Figure 6(a)** **and (b)** reveal that the product has a nanosheet appearance with a shrunken edges and irregular morphology structure at low temperature. However, ZnO nanosheet product gradually started to crumble and overlapped in a proportional relation with the increment of calcination temperature as shown in **figure 6** **(c) and (d)**.

**Figure 7:**
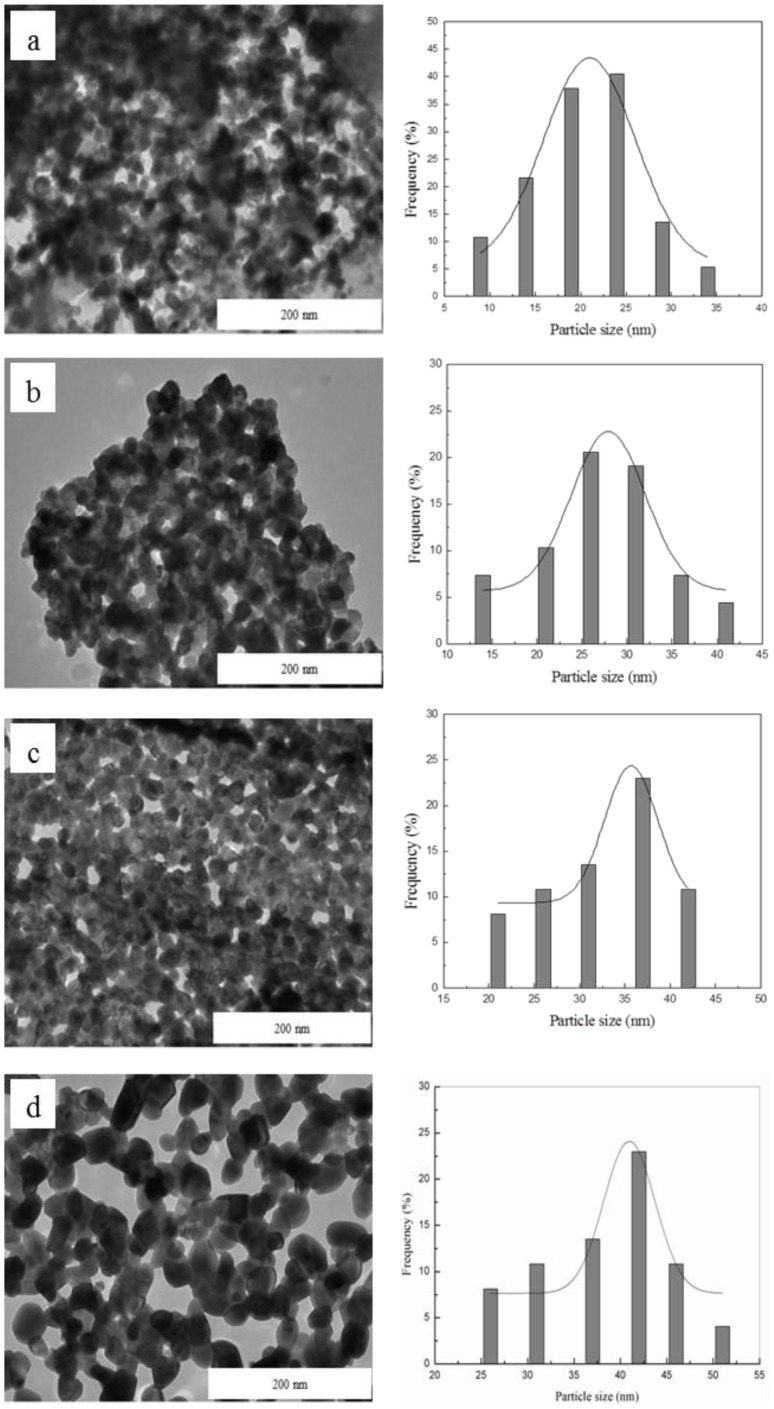
TEM images and particle size distribution of ZnO nanosheets at calcination temperature of (a) 500, (b) 550, (c) 600 and (d) 650°C.

Transmission electron microscopy (TEM) images were used to demonstrate the morphology, particle size and particles' size distribution of ZnO nanoparticles and are shown in **figure 7 (a), (b), (c) and (d)**. The obtained images show that the ZnO sample prepared by thermal-treatment method has exhibited uniform morphology and good particle size distribution. The average particle size at calcination temperatures of 500, 550, 600 and 650°C is about 23, 26, 30 and 38 nm, respectively, which were found to be in good agreement with the XRD measurements. These results indicate that the attained particle size has increased with increasing calcination temperature due to the fact that as the temperature went high, many neighbouring particles were prone to fuse together to form larger particle sizes by melting their surfaces [37].

### 6. Optical properties

Diffuse reflectance spectra are normally determined by a UV-visible spectrophotometer. The diffuse reflectance technique is capable of collecting the reflected light flux after interacting with the targeted sample. Then, the technique compares the reflected light fluxes coming from the surfaces of targeted sample and the standard reference sample.

**Figure 8:**
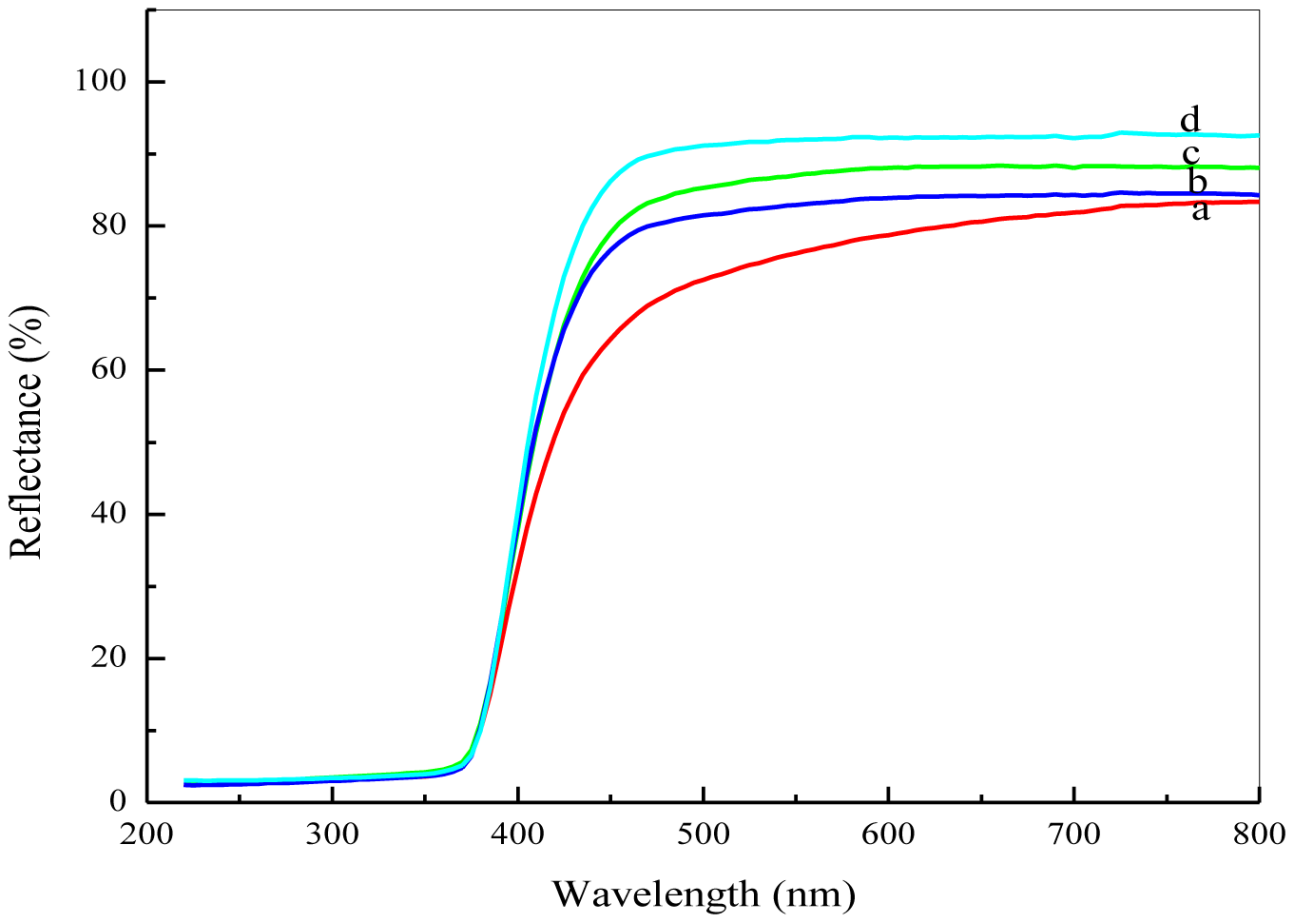
Reflectance of ZnO nanosheets calcined at different temperatures of (a) 500 (b) 550 (c) 600 (d) 650°C.

**Figure 9:**
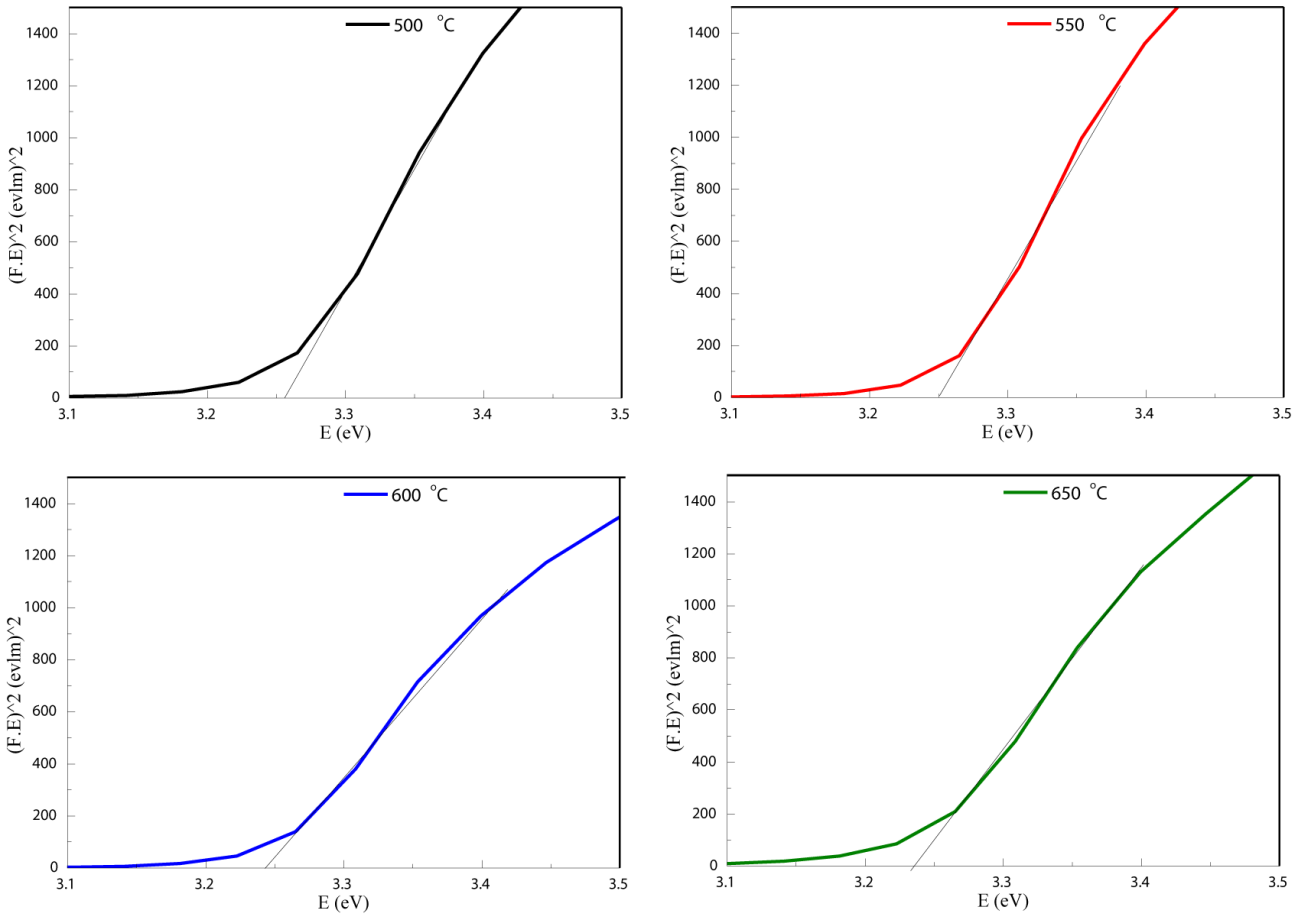
The method of extracting the band gaps of ZnO nanosheets calcined at different temperatures.

In order to investigate the calcination effect on the optical properties of ZnO nanosheets, the diffuse reflectance spectra, as shown in **figure 8**, were measured in the range of 200–800 nm at room temperature for all calcined samples. The optical band gap values for all samples calcined at different temperatures were determined from reflectance spectra using Kubelka-Munk equation;
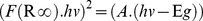
(2)where F(R_∞_) is the so-called remission parameter or Kubelka-Munk function, *hv* is the incident photon energy, *A* is a constant depending on the transition probability and the diffuse reflectance R_∞_, R_∞_ is the diffuse reflectance that is obtained from R_∞_ = R_sample_/R_standard_ [38]. The values of (F(R_∞_). *hv*)^2^ versus (*hv*) were plotted as illustrated in **figure 9**. Straight lines were drawn to fit the experimental band gap curves and were extended to intercept the (*hv*) axis in order to determine the optical band gap values of the ZnO nanosheets at different calcination temperatures. It was found that the optical band gap has decreased with increasing calcination temperature from 3.260 eV at 500°C to 3.23 eV at 650°C as shown in **Table 1**. A decrease in the energy band gap with increasing calcination temperatures is attributed to the increase in the particle size as well as to crystallinity improvement, as was evidenced by XRD analysis. It is believed that as the particle size increases, the number of atoms that form a particle also increase, which consequently render the valence and conduction electrons more attractive to the ions core of the particles and hence decreasing the band gap of the particles.

## Conclusions

ZnO nanosheets have been successfully synthesized by thermal-treatment method using only zinc nitrate as metal precursor, PVP as a capping agent and deionized water as a solvent. The calcination has enabled the removal of organic compounds and nitrate ions leaving a residue of pure crystalline ZnO nanosheets. The average particle size decreased from 23 to 38nm when increasing calcination temperature from 500 to 650°C, respectively. The band gap energy of ZnO nanosheets has been determined from the reflectance spectra and found decreasing from 3.260 eV at 500°C to 3.23 eV at 650°C. The thermal-treatment method is proven to be a simple and low cost method to synthesize ZnO nanosheets with high purity and also being friendly to the environment because of lack of chemical regents used and no toxic by-product effluents.
